# Morphological and molecular characterization of *Heterodera dunensis* n. sp. (Nematoda: Heteroderidae) from Gran Canaria, Canary Islands

**DOI:** 10.21307/jofnem-2020-098

**Published:** 2020-11-24

**Authors:** Phougeishangbam Rolish Singh, Gerrit Karssen, Marjolein Couvreur, Wim Bert

**Affiliations:** 1 Nematology Research Unit, Department of Biology, Ghent University, K.L. Ledeganckstraat 35, 9000, Ghent, Belgium; 2 National Plant Protection Organization, Wageningen Nematode Collection, P.O. Box 9102, 6700, HC, Wageningen, The Netherlands

**Keywords:** 18S, 28S, Canary Islands, *COI*, Cyst nematode, ITS, Gran Canaria, *Heterodera dunensis*, Plant-parasitic nematodes, *Schachtii*, Systematics, Taxonomy

## Abstract

*Heterodera dunensis* n. sp. from the coastal dunes of Gran Canaria, Canary Islands, is described. This new species belongs to the *Schachtii* group of *Heterodera* with ambifenestrate fenestration, presence of prominent bullae, and a strong underbridge of cysts. It is characterized by vermiform second-stage juveniles having a slightly offset, dome-shaped labial region with three annuli, four lateral lines, a relatively long stylet (27-31 µm), short tail (35-45 µm), and 46 to 51% of tail as hyaline portion. Males were not found in the type population. Phylogenetic trees inferred from D2-D3 of 28S, partial ITS, and 18S of ribosomal DNA and *COI* of mitochondrial DNA sequences indicate a position in the ‘*Schachtii* clade’.

The cysts forming nematodes of the genus *Heterodera* Schmidt, 1871 (Nematoda: Heteroderidae) are an economically important plant-parasitic nematode (PPN) group with a worldwide distribution and a broad host range causing prominent damages to the host plants ranging from stunted and reduced growth to wilting, chlorosis, and reduced root system (Perry et al., 2018; Sikora et al., 2018). The vermiform second-stage juveniles (J2) of this PPN migrate in the root system of a host plant to feed on the vascular cylinder where they become obese sedentary females; subsequently, following fertilization and egg production, these females turn into protective cysts of more or less lemon shape, housing numerous embryonated eggs. These eggs can remain viable for years inside the cysts, until favorable environmental conditions initiate hatching of the cysts to continue further life cycle (Subbotin et al., 2010; Perry et al., 2018).

Within this genus, 85 nominal species, eight species inquirendae, and a nomen nudum have been listed in a recent update by Handoo and Subbotin (2018). Using morphological and molecular characteristics, the species of this genus have been divided into nine groups, i.e., *Afenestrata*, *Avenae*, *Bifenestra*, *Cardiolata*, *Cyperi*, *Goettingiana*, *Humuli*, *Sacchari*, and *Schachtii.* Morphological characterization of *Heterodera* species is mainly done based on vulva-slit length, vulval cone fenestration, presence or absence of bullae and underbridge in female cysts, and stylet length, lateral field differentiation, tail length, and hyaline tail length in J2 (Subbotin et al., 2010). Since the last two decades, employing molecular data such as ITS and 28S of ribosomal DNA and *COI* gene of mitochondrial DNA to characterize *Heterodera* species has been a common practice, including DNA barcoding, phylogeny, and even phylogeography (Ferris et al., 1999; Toumi et al., 2013a; Subbotin et al., 2017, 2018).

Herein, we characterize *Heterodera dunensis* n. sp. discovered in a recent exploratory survey of PPN from Canarian coastal dunes of Gran Canaria in May 2019. The species characterization is done based on light microscopy (LM), scanning electron microscopy (SEM), and molecular information of ITS, 18S, and 28S of ribosomal DNA and *COI* of mitochondrial DNA.

## Materials and methods

### Nematode extraction and morphological analysis

A sandy soil sample was collected from around the root system of *Tetraena fontanesii* (Webb & Berthel.) Beier & Thulin, commonly known as Sea Grape or Canarian Bean-Caper. This halophilic succulent plant was growing on a dune (GPS coordinates: 27°44′19.11″ N; 15°35′0.3″ W), about 200 m away from the Maspalomas beach of Gran Canaria. Vermiform J2 was extracted from the sand using the modified Bearmann method (Whitehead and Hemming, 1965) and stored at 4°C during the course of analysis.

For collecting female cysts, sand dried at room temperature was mixed thoroughly in water using a spoon, and after letting the sand settle, floating female cysts were picked out using a fine brush.

Morphological study of J2 was done using both heat-relaxed and fixed specimens. Individual live nematodes were heat-relaxed in a drop of water on a glass slide and examined, photographed, and measured using an Olympus BX51 DIC Microscope (Olympus Optical, Tokyo, Japan), equipped with an Olympus C5060Wz camera and a drawing tube as described in the study of Singh et al. (2018). After recording morphological information, the specimens were recovered from the slide and their genomic DNA was extracted as described in the next section. The remaining J2 juveniles were concentrated in a drop of water in a glass embryo dish, followed by adding a few drops of freshly prepared Trump’s fixative [2% paraformaldehyde, 2.5% glutaraldehyde in 0.1 M Sorenson buffer (sodium phosphate buffer at pH = 7.3)]. The nematodes were then immediately heated in a microwave (700 watts) for about 4 sec, left to rest for 1 hr at room temperature and at 4°C for 24 hr, followed by gradually transferring to anhydrous glycerin to be mounted on glass slides as described in the study of Singh et al. (2018). Vulva cones of female cysts were cut in a drop of water under a stereomicroscope using a blade, and the cones were mounted in glycerol-gelatin (1:1) mix on glass slides. The mounted J2 juveniles and the female vulva cones of the cysts were studied and drawn using the above-mentioned camera- and drawing tube-equipped microscope. Illustrations were improved using Adobe Photoshop CS6 Version 13.0  x 64. For SEM, specimens fixed in Trump’s fixative were washed in 0.1 M phosphate buffer (pH = 7.3), and cysts were additionally sonicated for 8 min to remove any attached dirt, and dehydrated in a graded series of ethanol solutions, critical-point-dried with liquid CO_2_, mounted on stubs with carbon tabs (double conductive tapes), coated with gold of 25 nm, and photographed with a JSM-840 EM (JEOL) at 12 kV (Singh et al., 2018).

### Molecular analysis

Heat-relaxed nematodes after morphological analysis were recovered from temporary slides. Each individual nematode was cut into pieces in distilled water using a blade, and the pieces were transferred to a PCR tube with 20 µl of worm lysis buffer [50 mM KCl, 10 mM Tris at pH = 8.3, 2.5 mM MgCl_2_, 0.45% NP 40 (Tergitol Sigma), and 0.45% Tween 20]. DNA extraction from cysts containing embryonated eggs was also done for which an individual cyst was crushed and transferred in the PCR tube containing worm lysis buffer as mentioned. The PCR tubes were then incubated at −20°C (10 min) followed by adding 1 µl of proteinase K (1.2 mg/ml), incubation at 65°C (1 hr) and 95°C (10 min), and ending by centrifuging the mixture at 14,000 rpm for 1 min (Singh et al., 2018). PCR amplifications of partial ITS and 18S regions of ribosomal DNA were done using the primer pairs, Vrain2F: 5′-CTTTGTACACACCGCCCGTCGCT-3′/Vrain2R: 5′-TTTCACTCGCCGTTACTAAGGGAATC-3′ (Vrain et al., 1992) and SSU18A: 5′-AAAGATTAAGCCATGCATG-3′/SSU26R: 5′-CATTCTTGGCAAATGCTTTCG-3′ (Mayer et al., 2007) and with thermal profiles described in the study of Singh et al. (2018, 2019). For amplification of D2-D3 expansion segment of 28S of ribosomal DNA, two primer sets were used, the primer pair, 391: 5′-AGCGGAGGAAAAGAAACTAA-3′/501: 5′-TCGGAAGGAACCAGCTACTA-3′ was used as described in the study of Nadler et al. (2006) and D2A: 5′-ACAAGTACCGTGAGGGAAAGTTG-3′/D3B: 5′-TCCTCGGAAGGAACCAGCTACTA-3′ (Nunn, 1992) with the thermal profile from Singh et al. (2019). For the amplification of the *COI* region of mitochondrial DNA, the primer pair, JB3: 5′-TTTTTTGGGCATCCTGAGGTTTAT-3′/JB4.5: 5′-TAAAGAAAGAACATAATGAAAATG-3′ was used according to Bowles et al. (1992). The PCR products were enzymatically cleaned with alkaline phosphatase (1 U/ml) and exonuclease I (20 U/ml) for 15 min at 37°C followed by 15 min at 80°C and sent for sequencing at Macrogen (https://dna.macrogen.com), and contigs were made from the newly produced forward and backward sequences using Geneious Prime 2020.0.5 (https://www.geneious.com) and deposited in GenBank.

### Phylogenetic analysis

The phylogenetic relationships of the new species with other related species were analyzed based on the D2-D3, ITS, 18S, and *COI* sequences. Phylogenetic programs implemented in Geneious Prime 2020.0.5 were used. The obtained consensus contigs were subjected to BLAST search to check for closely related species on GenBank, and all the collected sequences for each gene fragment were aligned using MUSCLE alignment of Geneious Prime 2020.0.5 using default parameters, followed by manually trimming off of the poorly aligned ends. The best nucleotide substitution model of each gene alignment (see Figures) was determined by jModelTest 2.1.10. Bayesian phylogenetic analysis (MrBayes 3.2.6) was carried out using the selected models, analyses were run under 1 × 10^6^ generations (4 runs), and Markov chains were sampled every 100 generations, and 20% of the converged runs were regarded as burn-in (Huelsenbeck and Ronquist, 2001).

## Results

### Systematics

#### *Heterodera dunensis* n. sp.

**Figure 1:**
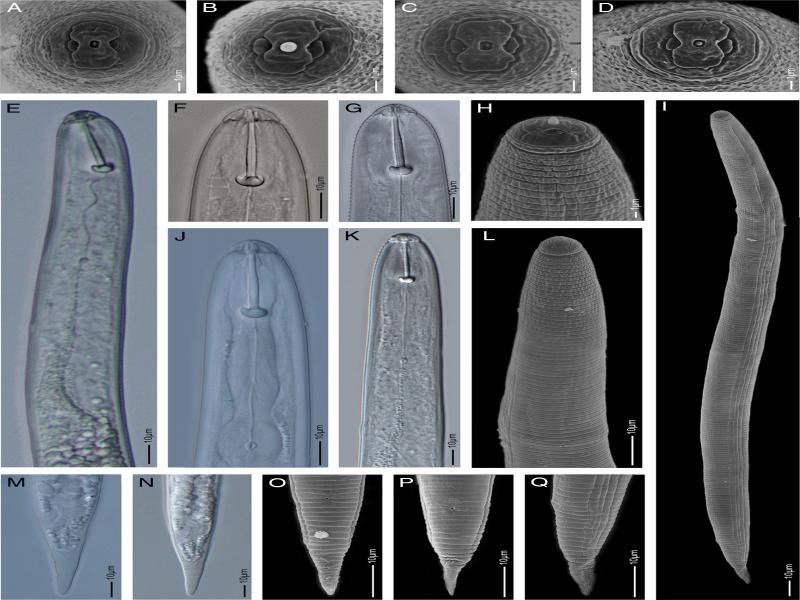
Light microscopy and scanning electron microscopy images of second-stage juveniles of *Heterodera dunensis* n. sp. A to D: *En face* view, E: Anterior part up to pharyngeal gland end, F to H: Labial region showing stylet and labial annuli, I: Total body, J to L: Anterior part showing median bulb, hemizonid, and secretory-excretory pore, M to Q: Tail region showing hyaline portion and anus.

**Figure 2:**
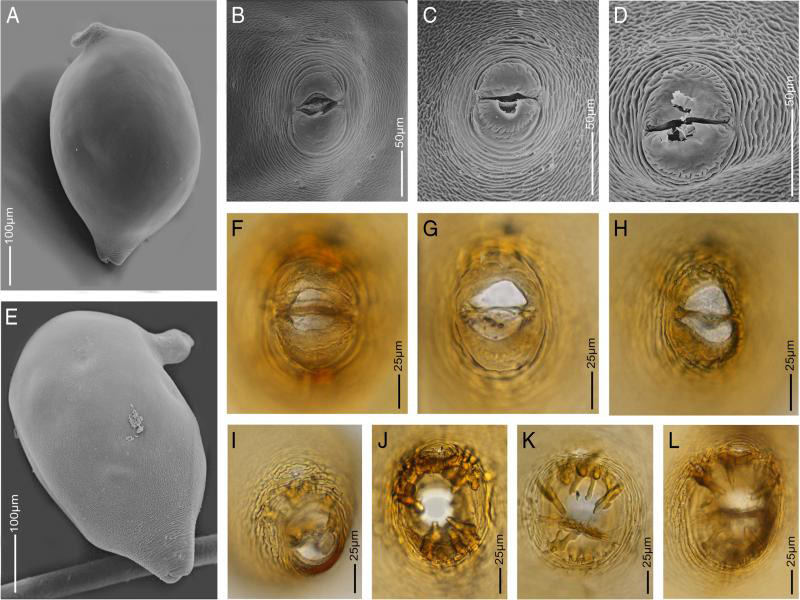
Light microscopy and scanning electron microscopy images of cysts and vulva cones of *Heterodera dunensis* n. sp. A, E: *In toto*, lemon-shape cysts showing protruded vulva cones and bent necks, B to D, F to H: Vulva cones showing cone fenestration and vulva slits, I to L: Vulva cones showing anus, bullae, and underbridge.

**Figure 3:**
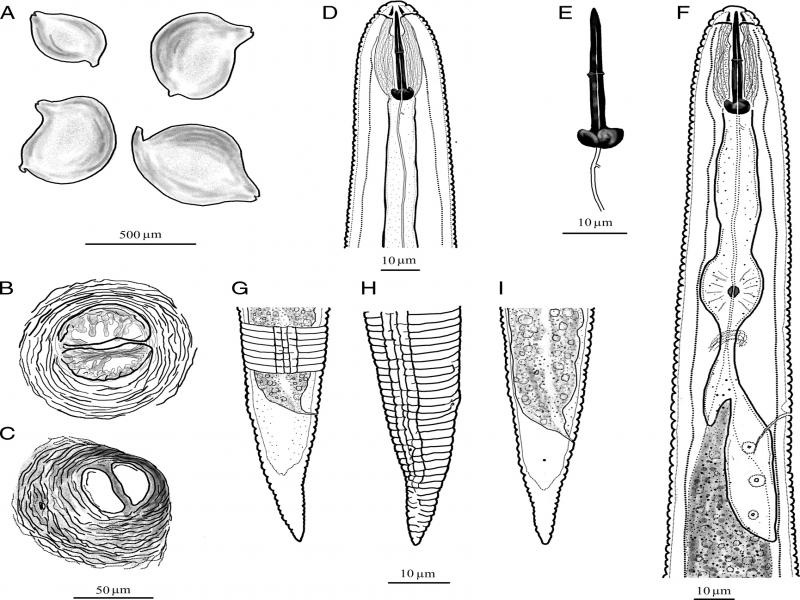
Line illustrations of cysts and second-stage juveniles (J2) of *Heterodera dunensis* n. sp. A: Whole cysts, B, C: Vulva cones showing cone fenestration, vulva slit, bullae, underbridge, and anus, D, F: Anterior part of J2 showing lip region, pharynx, hemizonid, and secretory excretory pore, E: Stylet of J2, G to I: Tail region of J2 showing lateral field differentiation, anus, phasmid, and tail hyaline portion.

**Figure 4:**
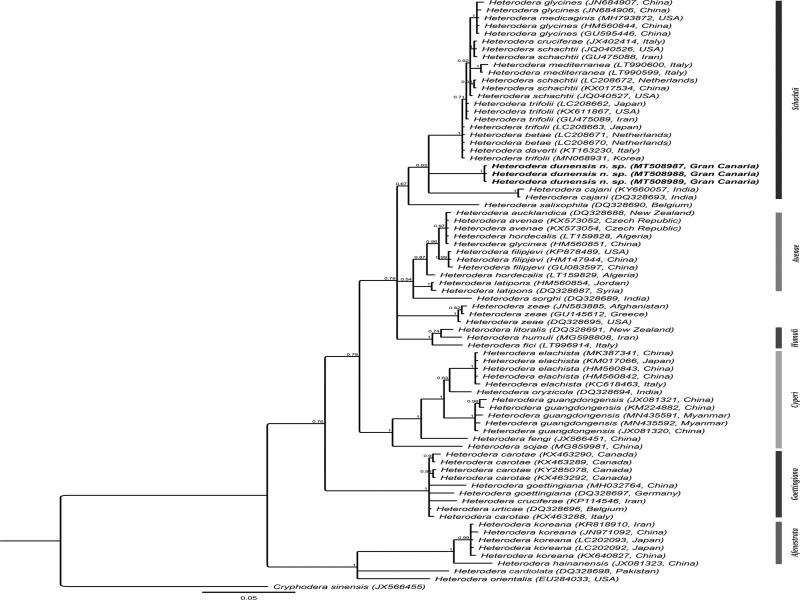
Phylogenetic relationships of *Heterodera dunensis* n. sp. with 33 known *Heterodera* species. Bayesian 50% majority-rule consensus tree as inferred from the analysis of D2-D3 of 28S rDNA sequences under GTR + G model. Posterior probabilities of more than 0.5 are given for appropriate clades.

**Figure 5:**
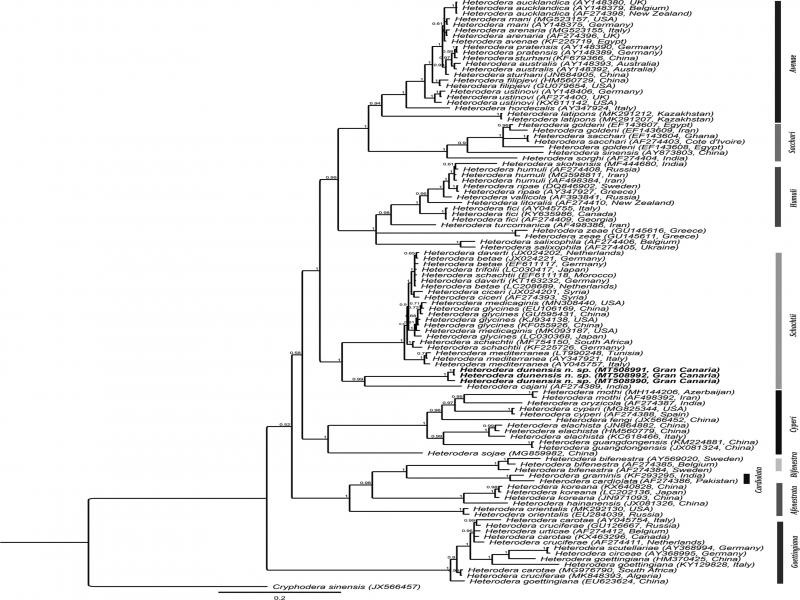
Phylogenetic relationships of *Heterodera dunensis* n. sp. with 54 known *Heterodera* species. Bayesian 50% majority-rule consensus tree as inferred from the analysis of partial ITS of rDNA sequences under GTR + I + G model. Posterior probabilities of more than 0.5 are given for appropriate clades.

**Figure 6:**
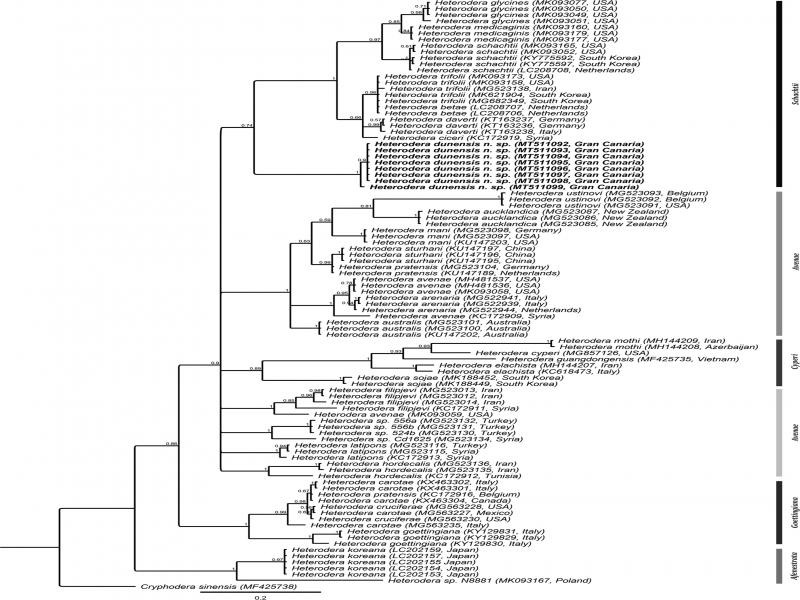
Phylogenetic relationships of *Heterodera dunensis* n. sp. with 29 known *Heterodera* species. Bayesian 50% majority-rule consensus tree as inferred from the analysis of *COI* of mtDNA sequences under GTR + I + G model. Posterior probabilities of more than 0.5 are given for appropriate clades.

Figures 1–6, Tables 1 and 2.

**Table 1.** Morphometric data of *Heterodera dunensis* n. sp. from fixed specimens mounted in glycerin, except for cysts that were not fixed.

Cysts	
*n*	21
Length without neck (*L*)	482 ± 75 (316-610)
Maximum cyst width (*W*)	360 ± 72.1 (241-478)
Neck length	110 ± 31.9 (55-180)
*L*/*W*	1.4 ± 0.2 (1.2-1.9)
Vulval area	
*n*	13
Fenestral length	42.1 ± 4.3 (37-51)
Mean semifenestral width	41.8 ± 5.2 (34-53)
Vulva bridge width	8.1 ± 1.6 (5.2-11)
Vulva slit length	52.1 ± 7.3 (35-63)
Underbridge length	63.4 ± 9.8 (44-83)
Underbridge width	18.2 ± 6.2 (8.8-29)
Vulva-anus distance	58.4 ± 5.7 (50-68)
Juveniles	
*n*	21
Body length (*L*)	471 ± 25.3 (426-520)
*a* = *L*/MBD	20.1 ± 1.4 (19-23)
*b* = *L*/Anterior end to pharynx-intestine junction	3.6 ± 0.4 (3.1-4.8)
*b*′ = *L*/Anterior end to the end of pharyngeal gland	2.7 ± 0.2 (2.4-3.1)
*c* = *L*/Tail length	11.6 ± 0.6 (11-12)
*c*′ = Tail length/ABD	33.6 ± 2.6 (27-39)
Stylet length	29.1 ± 1.1 (27-31)
Stylet knob width	6.4 ± 0.4 (5.9-7)
Lip region height	4.8 ± 0.3 (4.3-5.3)
Lip region width	9.1 ± 0.4 (8.6-10)
Dorsal gland opening from stylet base	3.7 ± 0.4 (3.2-4.4)
Anterior end to median valve	74.2 ± 2.4 (70-79)
Anterior end to secretory-excretory pore	109 ± 3.9 (101-117)
Anterior end to pharynx-intestine junction	131 ± 11.1 (103-155)
Anterior end to pharyngeal gland end	177 ± 9.2 (156-193)
Maximum body diameter (MBD)	23.5 ± 1.4 (21-26)
Anal body diameter (ABD)	14.1 ± 0.8 (13-16)
Tail length	40.7 ± 2.4 (35-45)
Hyaline tail length	20.6 ± 1.9 (16-23)

**Note:** All measurements are except percentage and ratio in μm and in the form: mean ± sd (range).

**Table 2.** Comparison of important characters of seventeen *Heterodera* species of the *Schachtii* group.

Character	Cyst length (*L*)	Cyst width (*W*)	*L*/*W* ratio	Fenestral length	Fenestral width	Vulval slit length	J2 body length	J2 stylet length	J2 tail length	J2 tail hyaline length	Hyaline% of the tail
*H. agrostis*	429-800	320-541	1.2-2.6	34-54	16-24	39-48	384-472	25-26	46-61	27-40	59-66
*H. betae*	830-878	455-518	1.6-1.9	44-55	38-43	48-57	547-607	29-31	70-74	38-42	54-57
*H. cajani*	448-670	209-422	1.4-2.1	27-65	25-40	43-55	420-519	22-27	42-52	23-31	55-60
*H. ciceri*	570-930	350-550	1.6-2.4	32-52	20-37	43-60	440-585	27-30	53-72	31-42	58-58
*H. daverti*	650-749	380-491	1.4-1.5	42-54	31-40	47-52	457-476	25-26	54-57	30-33	56-58
*H. dunensis n. sp.*	*316-610*	*241-478*	*1.2-1.9*	*37-51*	*34-53*	*35-63*	*426-520*	*27-31*	*35-45*	*16-23*	*46-51*
*H. galeopsidis*	576-797	408-556	1.4-1.5	41-50	31-38	39-50	485-553	26-28	61-75	35-40	57-53
*H. glycines*	474-709	327-535	1.3-1.7	34-58	16-41	38-50	386-471	21-23	39-51	22-30	56-59
*H. lespedezae*	678-719	371-522	1.4-1.8	43-59	35-41	45-47	457-481	24-25	54-56	26-30	48-54
*H. medicaginis*	568-728	364-570	1.4-1.5	39-55	30-40	39-55	417-512	24-26	41-60	22-33	54-55
*H. mediterranea*	430-690	240-570	1.2-1.6	38-45	37-42	42-48	360-430	25-27	38-45	19-26	50-58
*H. rosii*	537-1173	403-634	1.0-1.7	48-65	40-45	45-59	430-662	27-34	58-77	37-45	64-58
*H. schachtii*	768-815	512-529	1.5-1.6	35-38	25-31	41-44	436-489	25-26	45-49	24-27	53-55
*H. sonchophila*	732-1032	381-616	1.6	37-58	29-50	42-56	437-492	24-27	47-56	26-30	55-54
*H. spiraeae*	467-861	283-566	1.1-1.7	33-60	20-45	39-45	371-446	22-23	39-49	21-27	54-55
*H. swarupi*	520-700	320-475	1.6-2.1	45	35	41	400-440	21-23	39-54	20-29	51-54
*H. trifolii*	608-841	341-536	1.3-1.8	43-53	33-44	40-53	492-613	25-28	60-72	32-37	53-51

**Note:** The measurements of the new species are shown in bold. All measurements are in µm and presented as range.

*Descrip* *tion*

Cyst: Body typical lemon shape to sometimes ovoid shape with protruding prominent neck and vulva. Neck regularly bent. Cysts wall light to medium brown in color with irregular zig-zag pattern on surface. Fenestration ambifenestrate. Vulva cone dome-shaped with sub terminal anus. No egg-sac observed. Vulva slit longer than fenestral length. Bullae prominent, medium brown in color, variable shape, in some cysts commonly finger-like or elongated, irregularly distributed at the periphery of vulva cone slightly above underbridge level. Underbridge furcated with central thickening, prominent in young cysts, breaks down in older cysts. Cysts containing 100-200 eggs.

J2: Body slender, tapering posteriorly. Labial region slightly offset, dome-shaped with two clear incisure under LM appearing as three lip annuli, second annule wider than the other two. *En face* showing oral disc fused with submedial sectors, well-separated lateral lip sectors and rectangular to square-shaped stoma opening. Lateral field with four longitudinal incisures forming three bands, outer two bands slightly wider than inner. All bands irregularly areolated, sometimes with incomplete areolation. Stylet robust, 27-31 µm long, with large rounded strongly anteriorly projecting knobs. Pharynx well-developed, ca one-third of body length with well-developed median bulb, valves and glands overlapping intestine ventrally. Nerve ring encircling isthmus. Hemizonid distinct, about two cuticular annuli long, just above secretory-excretory (SE) pore opening. SE pore at ca one-fourth of body length from anterior end. Tail 35-45 µm long, tapers gradually to a rounded terminus, hyaline region ca 50% of tail length. Phasmid opening small, roughly halfway between anus and start of hyaline tail part.

Male:

Not found.

#### Diagnosis and relationships

*Heterodera dunensis* n. sp. is characterized by moderate-sized J2 of 0.43 to 0.52 mm long, lateral field with four lines, the inner band slightly smaller than the outer two bands, and all bands with irregular areolation throughout the length; a relatively long J2 stylet of 27 to 31 µm with anteriorly projected knobs, a relatively short tail of 35 to 45 µm in length, small rounded phasmids, and tail hyaline part usually ca 50% of the tail; cyst ovoid to regularly lemon-shaped, ambifenestrate, the presence of prominent finger-like bullae, and a strong underbridge.

This new species belongs to the *Schachtii* group that comprises sixteen *Heterodera* species, i.e., *Heterodera agrostis* Kazachenko, 1993; *Heterodera betae* Wouts, Rumpenhorst and Sturhan, 2001; *Heterodera cajani* Koshy, 1967; *Heterodera ciceri* Vovlas, Greco and Di Vito, 1985; *Heterodera daverti* Wouts and Sturhan, 1978; *Heterodera galeopsidis* Goffart, 1936; *Heterodera glycines* Ichinohe, 1952; *Heterodera lespedezae* Golden and Cobb, 1963; *Heterodera medicaginis* Kirjanova in Kirjanova and Krall, 1971; *Heterodera mediterranea* Vovlas, Inserra and Stone, 1981; *Heterodera rosii* Duggan and Brennan, 1966; *Heterodera schachtii* A. Schmidt, 1871; *Heterodera sonchophila* Kirjanova, Krall and Krall, 1976; *Heterodera spiraeae* Kazachenko, 1993; *Heterodera swarupi* Sharma, Siddiqi, Rahaman, Ali and Ansari, 1998; and *Heterodera trifolii* Goffart, 1932. They are all similar in having J2 with a lateral field with four lines, with more or less anteriorly projected stylet knobs; cysts presented with ambifenestrate fenestration, the presence of prominent bullae, and a strong underbridge. *Heterodera dunensis* n. sp. can be easily differentiated from other members of *Schachtii* group based on J2 with a long stylet, short tail, and a short hyaline region. It differs from *H. agrostis, H. daverti, H. glycines, H. lespedezae, H. medicaginis, H. schachtii, H. spiraeae,* and *H. swarupi* in having a distinctly longer J2 stylet of 29 µm (27-31 µm) vs stylet length always shorter than 27 µm.

This new species can also be easily separated from all the members of the group, except *H. mediterranea* by its shorter J2 tail length of 41 µm (35-45 µm) vs always above 45 µm on average (38-77 µm) and a shorter hyaline tail part of 21 µm (16-23 µm) vs always above 23 µm on average (20-45 µm) in the other species. *Heterodera dunensis* n. sp. is morphologically closest to *H. mediterranea* with several overlapping morphometrics, such as the dimension of cyst cone fenestration and length of vulva slit, the length of J2 tail and hyaline part, but differs from this species in the J2 body length (426-520 vs 360-430 µm), by a slightly longer J2 stylet (27-31 vs 25-27 µm), presence vs absence of finger-like bullae and central thickening of an underbridge in its respective cysts.

*Heterodera dunensis* n. sp. is also based on D2-D3, ITS, 18S, and *COI* sequences clearly different from all known species, see below.

#### Molecular characterization

##### D2-D3 of 28S rDNA

Three D2-D3 sequences (MT508987-MT508989) of 987-1039 bp were produced without intraspecific sequence variation. The closest available sequence on GenBank was MK292129 of *H. glycines* with 95.9% similarity (43 out of 1039 bp differences). The D2-D3 alignment of 750 bp long consisted of 75 *Heterodera* sequences of 33 species and a *Cryphodera sinensis* sequence (JX566455) as the outgroup. The resulting D2-D3 tree revealed an unresolved position of *H. dunensis* n. sp. in a clade (PP = 0.93) comprising eight members of *Schachtii* group, i.e. *H. glycines*, *H. medicaginis*, *H. schachtii*, *H. mediterranea*, *H. trifolii*, *H. betae*, *H. daverti*, and *H. cajani*.

##### ITS of rDNA

Three partial ITS sequences (MT508990-MT508992) of 747-1025 bp were produced with intraspecific sequence variation of only one bp. The closest available sequence was LC030416 of *H. trifolii* with 84.6% sequence similarity (150 out of 971 bp differences). The ITS alignment was 1514 bp long and consisted of 105 *Heterodera* sequences of 54 species and a *Cryphodera sinensis* sequence (JX566457) as the outgroup. In the inferred ITS tree, *H. dunensis* n. sp. occupies a well-supported sister relationship with *H. cajani* (PP  =  0.99) within a maximally supported clade of other members of *Schachtii* group, namely *H. daverti*, *H. betae*, *H. trifolli*, *H. schachtii*, *H. ciceri*, *H. medicaginis*, *H. glycines*, and *H. mediterranea.*

##### 18S of rDNA

 Three partial 18S sequences (MT509422-MT509424) of 806-845 bp were generated without intraspecific sequence variation. The sequences were closest to EU306357 of *H. koreana* with 98.9% similarity (9 out of 845 bp differences). The resulting phylogenetic tree revealed an unresolved position of several species, including *H. dunensis* n. sp., and is, therefore, not provided.

##### *COI* of mtDNA

Eight *COI* sequences (MT511092-MT511099) of 309-405 bp were generated without intraspecific sequence variation. The closest sequence available on GenBank was MN311179 of *H. medicaginis* with 87.6% similarity (46 out of 370 bp difference). A *COI* sequence alignment of 418 bp long was made consisting of 90 sequences from 29 *Heterodera* species, including the new species, five unidentified sequences, and a *Cryphodera sinensis* sequence (MF425738) as the outgroup. From the phylogenetic tree inferred, *H. dunensis* n. sp. formed a poorly supported sister relationship (PP = 0.74) with a clade consisting of *H. glycines*, *H. medicaginis*, *H. schachtii*, *H. trifolii*, *H. betae*, *H. daverti*, and *H. ciceri*.

##### Etymology

The species epithet refers to the coastal dunes, the type locality where this new species was found.

##### Type host and locality

*Heterodera dunensis* n. sp. was recovered from the rhizosphere of the halophilic host plant, *Tetraena fontanesii* (Webb & Berthel.) Beier & Thulin, growing on a dune, roughly 30 cm high, about 200 m inland of Maspalomas beach of Gran Canaria; GPS coordinates: 27°44′19.11″ N; 15°35′0.3″ W.

##### Type material

Holotype J2 and seven J2 paratypes in two slides, two cyst vulval cones, and two whole cysts in separate slides were deposited at the National Plant Protection Organization, Wageningen Nematode Collection, Wageningen, The Netherlands (WaNeCo). Six paratype J2 and three cyst vulval cones in two slides were submitted to the Ghent University Museum, Zoology Collections, Belgium. Additionally, five J2 paratypes and two cyst vulval cones were also deposited at the UGent Nematode Collection (slide UGnem-189-190) of the Nematology Research Unit, Department of Biology, Ghent University, Belgium.

## Discussion

*Heterodera dunensis* n. sp. is easily distinguishable from other *Heterodera* species and from all other members of *Schachtii* group by both morphology and molecular data (D2-D3, ITS, 18S, and *COI* sequences). The obtained phylogenetic trees revealed a consistent phylogenetic position of the new species always forming a clade together with other members of the *Schachtii* group. The ITS tree provided slightly better-resolved phylogenetic relationships among different *Heterodera* species compared to the D2-D3 and the COI trees, while the 18S tree had an inferior resolution.

*Heterodera dunensis* n. sp. was present in a Canarian dune sample with a moderately large population of only J2 and cysts together with very few saprophytic nematodes and no other PPN species. A resampling in search of males from the type location and an attempt to culture the species at NPPO, Wageningen green house did not succeed. Coastal regions are a relatively common habitat for *Heterodera* spp. To our knowledge, nine *Heterodera* spp. have been reported from similar habitats around the world, namely *Heterodera arenaria* Cooper, 1955, parasitizing on marram grass (*Ammophila arenaria*) on mobile sand dunes from several coastal sites in the United Kingdom and the Netherlands (Robinson et al., 1996; Brzeski, 1998); *Heterodera aucklandica* Wouts and Sturhan, 1995 associated with meadow rice grass (*Microlaena stipoides*) in Auckland, New Zealand (Wouts and Sturhan, 1995); *Heterodera hordecalis* Andersson, 1975 on marram grass in the Netherlands (Van der Putten and Van der Stoel, 2006); *Heterodera leuceilyma* Di Edwardo and Perry, 1964 from the coastal regions of Florida parasitizing on St Augustine grass (*Stenotaphrum secundatum*) (Di Edwardo and Perry, 1964); *Heterodera litoralis* Wouts and Sturhan, 1996 associated with a succulent plant, beaded glasswort (*Sarcocornia quingueflora*) in South Island, New Zealand (Wouts and Sturhan, 1996); *H. mediterranea* associated with a woody plant, lentisc (*Pistacia lentiscus*) on The Adriatic coast of Southern Italy (Vovlas et al., 1981); *Heterodera pratensis* Gäbler, Sturhan, Subbotin and Rumpenhorst, 2000 in a pasture near the coast of the Baltic Sea at Lindhöft, Germany (Gäbler et al., 2000); *Heterodera riparia* (Kazachenko, 1993) Subbotin, Sturhan, Rumpenhorst and Moens, 2003 associated with couch grass, *Elymus repens* (L.) from along the coast of Olga Bay, the coast of Okhot Sea, Kamchatka region of Russia (Subbotin et al., 2003), and *Heterodera salixophila* Kirjanova, 1969 parasitizing roots of the willow tree, *Salix purpurae* from the shores of Kurdish Bay, Baltic Sea, Kaliningrad region of Russia (Kirjanova, 1969).

This new species was found associated with *Tetraena fontanesii* (Sea Grape or Canarian Bean-Caper), a succulent plant with a limited distribution, in Canary Islands and some parts of West Africa. To the best of our knowledge, no report of an association of PPN with this host has been made before. Sampling in similar habitats should reveal if *H. dunensis* n. sp. is endemic for the Canarian islands and to what extent it is host-specific.
